# Ultrafast signatures of magnetic inhomogeneity in Pd_1−_*_x_*Fe*_x_* (*x* ≤ 0.08) epitaxial thin films

**DOI:** 10.3762/bjnano.13.74

**Published:** 2022-08-25

**Authors:** Andrey V Petrov, Sergey I Nikitin, Lenar R Tagirov, Amir I Gumarov, Igor V Yanilkin, Roman V Yusupov

**Affiliations:** 1 Kazan Federal University, Kremlyovskaya 18, Kazan, Russia; 2 Zavoisky Physical-Technical Institute, FRC Kazan Scientific Centre of RAS, Sibirsky trakt 10/7, Kazan, Russia

**Keywords:** magnetic inhomogeneities, PdFe alloy, thin epitaxial films, time-resolved magneto-optical Kerr effect, time-resolved optical spectroscopy

## Abstract

A series of Pd_1−_*_x_*Fe*_x_* alloy epitaxial films (*x* = 0, 0.038, 0.062, and 0.080), a material promising for superconducting spintronics, was prepared and studied with ultrafast optical and magneto-optical laser spectroscopy in a wide temperature range of 4–300 K. It was found that the transition to the ferromagnetic state causes a qualitative change of both the reflectivity and the magneto-optical Kerr effect transients. A nanoscale magnetic inhomogeneity of the ferromagnet/paramagnet type inherent in the palladium-rich Pd_1−_*_x_*Fe*_x_* alloys reveals itself through the occurrence of a relatively slow, 10–25 ps, photoinduced demagnetization component following a subpicosecond one; the former vanishes at low temperatures only in the *x* = 0.080 sample. We argue that the 10 ps timescale demagnetization originates most probably from the diffusive transport of d electrons under the condition of nanoscale magnetic inhomogeneities. The low-temperature fraction of the residual paramagnetic phase can be deduced from the magnitude of the slow reflectivity relaxation component. It is estimated as ≈30% for *x* = 0.038 and ≈15% for *x* = 0.062 films. The minimal iron content ensuring the magnetic homogeneity of the ferromagnetic state in the Pd_1−_*_x_*Fe*_x_* alloy at low temperatures is about 7–8 atom %.

## Introduction

Superconductor-based technologies are promising for exaflop-scale supercomputing, big-data processing, artificial intelligence, and neuromorphic computing [1–7]. The highlight features of superconducting data processing techniques, for example, RSFQ logic [1–9], are the high speed and unprecedental energy efficiency [2–3,10–13]. Superconducting spintronics is a branch of superconducting electronics, the key components of which are thin-film magnetic Josephson junctions (MJJs), which include layers of superconductors (S), ferromagnets (F) and insulators (I) [1–3,14–15]. The use of MJJs considerably reduces the energy consumption, the number of Josephson junctions, and the number of interconnects in superconducting digital circuitries [16], ensuring wide operation margin tolerances and low bit-error rates [17–18].

To realize the full functionality of superconducting digital circuits, several kinds of MJJ-based devices are required, including logic gates [19–23], programmable logics [16], non-dissipative biasing [1], and random access and cache memories [17,24–28]. From the fabrication point of view, it is strongly desirable to utilize a universal tunable ferromagnetic material for every application. Among several candidates [1–3], palladium-rich Pd_1−_*_x_*Fe*_x_* alloys look attractive because of the noble-metal base robust against deterioration and the possibility to tune the magnetic properties of Pd_1−_*_x_*Fe*_x_* alloy films by varying the iron content *x* and the preparation conditions [29–30]. Moreover, attempts have been made to use this material (with low iron concentrations of *x* = 0.01–0.03) for MJJ memory applications [1,14–15,24,31–32]. However, these studies faced the problems of small critical current and temporal instability of magnetic properties [33]. On the one hand, nanoscale magnetic inhomogeneities are inherent in disordered Pd_1−_*_x_*Fe*_x_* alloys with a high palladium content, on the other hand, these inhomogeneities are extremely undesirable in MJJs. Indeed, within the frame of the percolation model of ferromagnetism in Pd_1−_*_x_*Fe*_x_* alloys with *x* < 0.1 [34–35], magnetic inhomogeneities cause spin-flip and pairing wave function damping, thus, reducing the magnitude of the Josephson critical current. Small-scale inhomogeneities are difficult to detect with either conventional neutron-scattering methods [34] or with the stationary magneto-optical Kerr/Faraday effect and ferromagnetic resonance techniques (the latter two, because of the large scale, yield volume-averaged signals). Resonant magnetic small-angle X-ray scattering applied to Pd_1−_*_x_*Fe*_x_* alloy films with *x* = 0.03–0.07 revealed static magnetic fluctuations on the lateral scale of about 100 nm attributed to the magnetic domain structures of the films [36]. Smaller-scale fluctuations, due to intrinsic disorder in the alloy composition, still remain unexplored.

Finding a way to achieve magnetic uniformity in Pd_1−_*_x_*Fe*_x_* down to the atomic scale is a challenge. One of the options is the selection of the concentration range of iron at which the alloy would become magnetically homogeneous. This requires a method for detecting magnetic inhomogeneities, preferably with the possibility of being applied to thin films. We propose the use of ultrafast, time-resolved optical and magneto-optical spectroscopy methods for probing magnetic inhomogeneities in thin films. Individual constituents can be characterized by specific relaxation components that can be used to detect magnetic inhomogeneities and track their evolution. In addition, the peculiarities of the magnetization dynamics in magnetically inhomogeneous systems themselves are of interest.

In our recent work, using the example of a thin epitaxial film of Pd_0.94_Fe_0.06_, it was demonstrated [37] that the dynamics of the reflection coefficient and the angle of rotation of the polarization plane in magneto-optical Kerr effect (MOKE) measurements after a photoexcitation with femtosecond light pulses contain components whose temperature dependence correlates with that of the spontaneous magnetization. It was argued that such responses can serve as a source of information on magnetic inhomogeneities. In this work, we extend the series of Pd_1−_*_x_*Fe*_x_* films to a wider concentration range, confirm the correlation of the ultrafast responses with the magnetic properties of the system, and determine the minimum iron concentration in the alloy that ensures magnetic homogeneity at low temperatures. We discuss the findings in the frame of a model in which ferromagnetic (FM) and paramagnetic (PM) regions coexist, with the latter collapsing upon an increase of the iron content.

## Experimental

The samples for the studies were thin epitaxial films of Pd_1−_*_x_*Fe*_x_* with a nominal iron content of *x* = 0 (pure Pd), 0.038, 0.062, and 0.080 grown on single-crystal MgO(001) substrates by molecular beam epitaxy (MBE). The films were 20 nm thick, continuous, and smooth monocrystalline layers. The MBE equipment provided uniformity of the film thickness within 3% on the 1″ lateral size. The film composition *x* was measured in situ using X-ray photoelectron spectroscopy (all from SPECS, Berlin) with a nominal accuracy of 0.1%. Details of the synthesis and characterization of the samples used in the work can be found in our previous papers [29–30]. The Curie temperatures for the samples with *x* = 0.038, 0.062, and 0.080 were ≈120 K, ≈177 K, and ≈210 K, respectively.

The optical experiments were carried out in a pump–probe arrangement with a Legend-USP regenerative amplifier from COHERENT used as a light sourse in a similar way as described in [37]. The pulse repetition rate was 970 Hz, the central wavelength was 800 nm, and the duration was 40 fs. Excitation of the samples was performed by the pump light with a wavelength of 400 nm (second harmonic) and the properties were probed at 800 nm. The pump and probe beams were focused at the sample into the spots with diameters of 0.5 mm and 0.1 mm, respectively. Energy densities of the pump and the probe were 1 mJ/cm^2^ and 50 μJ/cm^2^, and the incidence angles were ≈2° and ≈18°.

The relaxation of the electronic subsystem was monitored by the relative change in the reflection coefficient (∆*R*/*R*). Ultrafast dynamics of the magnetization was analyzed by the deviation of the angle of rotation of the polarization plane of the probing light from the equilibrium in longitudinal MOKE measurements. MOKE reveals itself, in general, in a rotation of the polarization plane and an ellipticity of linearly polarized light on its reflection from a magnetized medium. Macroscopically, it originates from an occurrence of the finite non-diagonal components of the dielectric permittivity tensor of a medium proportional to its magnetization. Therefore, any of the real θ_K_ (rotation angle) or imaginary η_K_ (ellipticity) parts of the complex Kerr angle Θ_K_ = θ_K_ + *i*η_K_ provide a measure of the magnetization of a medium. An ability to track modifications of these quantities on an ultrafast time scale allows for the study of the magnetization dynamics. In our experiments, the probing light reflected from the sample passed through a Wollaston prism dividing the beam into two orthogonally polarized components. The intensities of these two components were detected by Hamamatsu S2386-5K silicon photodiodes. The difference signal from the output of the photodiodes was used to determine the rotation angle Δθ *= f*(Δ*t*), and the sum signal was used to measure the dynamics of the reflection coefficient Δ*R*/*R = g*(Δ*t*). To extract the magnetic contribution Δθ_Κ_ to the rotation of the polarization plane Δθ, which partially can originate from the pump-induced anisotropy, the responses were measured at two oppositely applied magnetic fields +*H* and −*H*. In this case, the contribution odd with respect to the sign of the field Δθ_Κ_ = [Δθ(+*H*) − Δθ(−*H*)]/2 has a magnetic nature.

To perform measurements at temperatures from 4.2 to 300 K, the films under study were mounted to the cold finger of the Janis ST-500 helium-flow cryostat. Permanent NdFeB magnets were fixed there, creating a magnetic field directed along the easy axis of the thin film in its plane with a magnitude of 470 Oe at room temperature. This field strength ensures a uniformly magnetized state of the film since the coercive field of the studied samples does not exceed 25 Oe. The sample temperature was set and maintained using a Lakeshore 335 temperature controller with an accuracy of 0.1 K.

## Results

**Figure 1:**
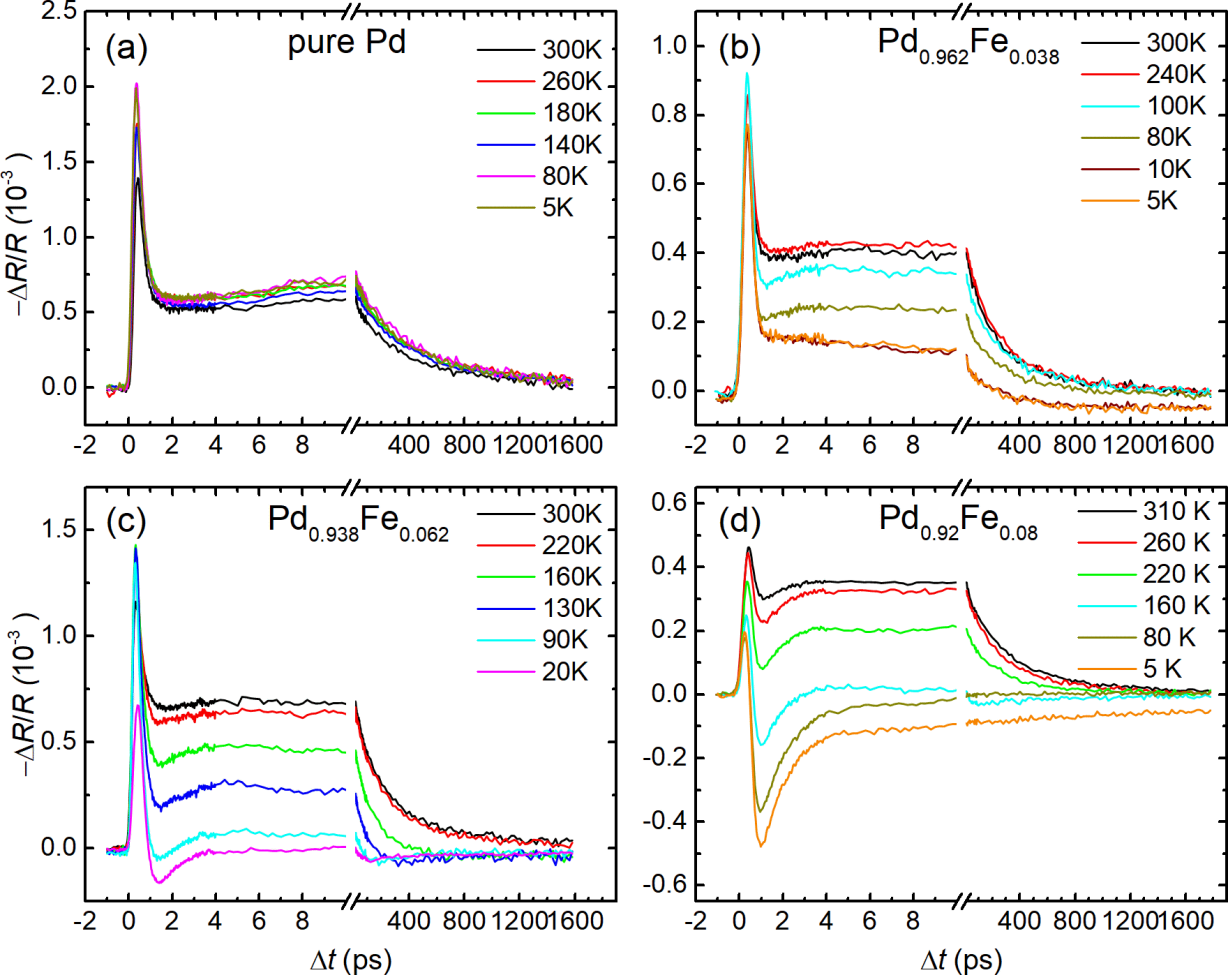
Temperature evolutions of the reflectivity transients of Pd_1−_*_x_*Fe*_x_* alloy thin epitaxial films for compositions with *x* = 0 (a), 0.038 (b), 0.062 (c), and 0.080 (d).

Figure 1 shows the dependency of the reflectivity normalized to the equilibrium value on the delay between the pump and the probe pulses of the four studied samples and its variation with temperature in the range of 5–300 K. In general, the responses of the pure palladium film change very slightly with temperature. The addition of the iron dopant leads to a development of a temperature dependence of Δ*R*/*R*(Δ*t*) responses, both qualitative (the appearance of new relaxation components) and quantitative (changes in their amplitudes and time constants). While two decaying exponents are sufficient to describe the relaxation of the reflection coefficient of the Pd and Pd_0.962_Fe_0.038_ films at the lowest temperature, a minimum of four is required for the Pd_0.94_Fe_0.06_ film and only three for Pd_0.92_Fe_0.08_. Thus, with an increase in the iron concentration *x* in a Pd_1−_*_x_*Fe*_x_* system, the photoinduced dynamics of the electronic subsystem changes from a relatively simple to a much more complex one; subsequently, the character partially simplifies again.

In quantitative terms, in the general case, the relaxation response can be described by the sum of four decaying exponents, two fast and two relatively slow ones, with one positive and one negative amplitude in each pair:


[1] $$-\Delta R/R\left(\Delta t\right)=A_{\text{f}}e^{-\Delta t/\tau_{\text{f}}^{A}}+A_{\text{s}}e^{-\Delta t/\tau_{\text{s}}^{A}}-B_{\text{f}}e^{-\Delta t/\tau_{\text{f}}^{B}}-B_{\text{s}}e^{-\Delta t/\tau_{\text{s}}^{B}}.$$

A significant difference in the values of the characteristic times for the fast and slow components makes it possible to fit them separately, which improves the accuracy of the parameter determination.

To describe the relaxation of the reflectivity of a palladium film, Figure 1a, the first two terms in Equation 1 are sufficient. The fast component with an amplitude *A*_f_ has a decay time 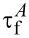 = 0.24 ± 0.02 ps. The lifetime of the second, slow component with the amplitude *A*_s_ is 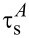 = 410 ± 10 ps. Figure 1b–d shows similar dynamics of the reflectance for three films with iron contents of 3.8, 6.2 and 8.0 atom %. At room temperature, the behavior of the responses for the films with 3.8 and 6.2 atom % of iron is similar to the responses obtained from the pure Pd film. The abovementioned fast component for these films has approximately the same lifetime, ≈0.3 ps. The lifetime of the slow component in the samples with 3.8, 6.2, and 8.0 atom % of iron is 240 ± 10, 210 ± 10, and 290 ± 10 ps, respectively. However, with an increase in the iron concentration, at times up to ca. 10 ps, an additional fast exponential decaying component appears. This component is opposite in sign to those given above. The main feature of these responses is their strong temperature dependence. At temperatures above the Curie temperature of the samples, they are not detectable. However, on cooling, starting from the Curie temperature, the Δ*R*/*R*(Δ*t*) responses increase sharply. The amplitude of the fast negative component increases in absolute value. Also, both the amplitude and the relaxation time of the slow positive component decrease. At temperatures of 90 and 160 K, another slow negative component appears in the samples with 6 and 8 atom % of iron, respectively. Its relaxation time is about 1 ns. The amplitude of this component is one order of magnitude smaller than the amplitudes of the other components.

**Figure 2:**
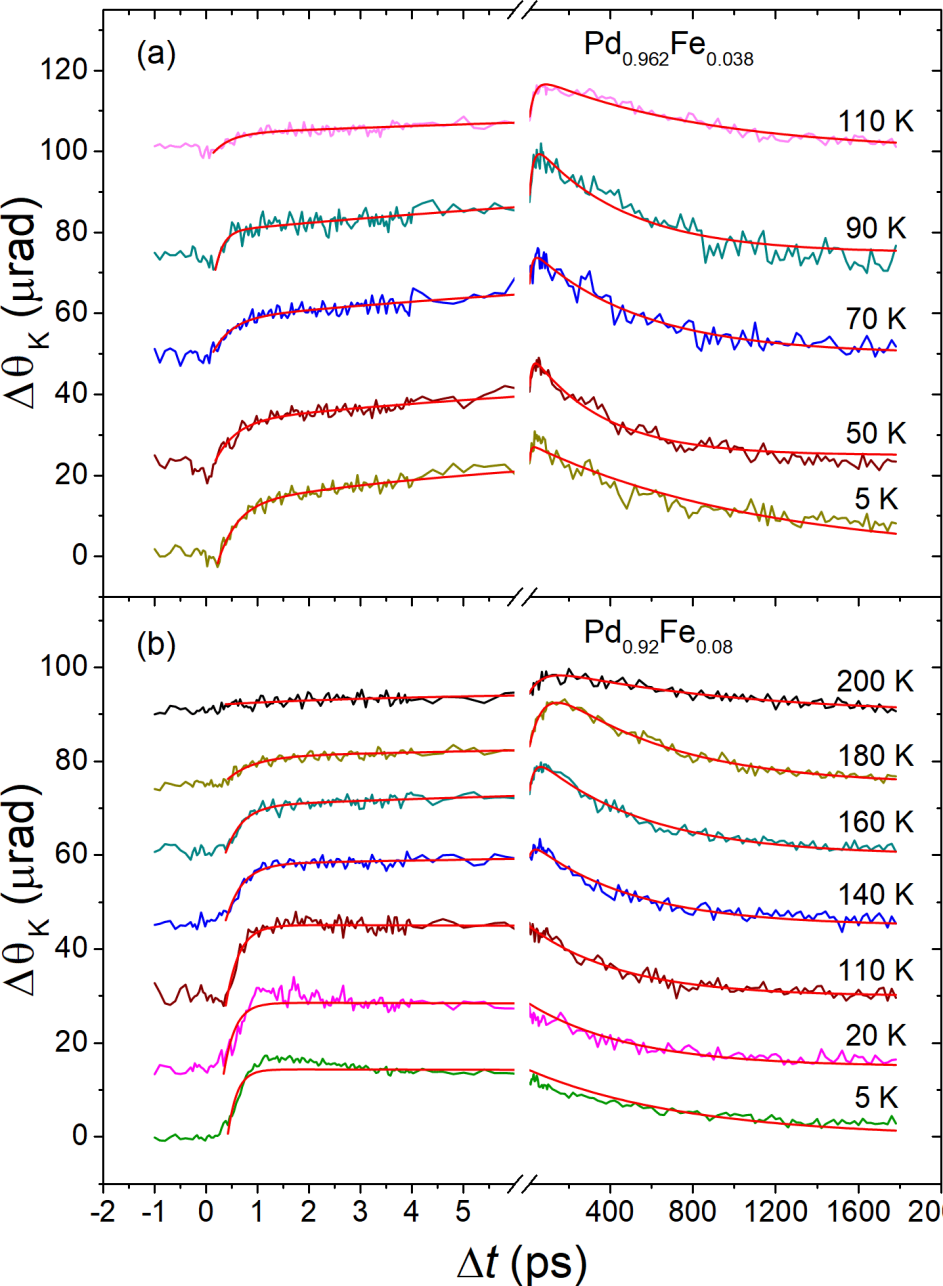
Temperature evolution of the time-resolved magneto-optical Kerr angle transients for the Pd_0.962_Fe_0.038_ (a) and Pd_0.92_Fe_0.08_ (b) epitaxial thin films at *T* < *T*_C_; red solid lines are the results of fits with Equation 2.

Figure 2 shows temperature dependency of the ultrafast dynamics of magnetization. The data are presented here for the films with *x* = 0.038 and *x* = 0.080; for the sample with *x* = 0.062, the responses can be found in [37]. Photoinduced demagnetization and the recovery are observed only at *T* < *T*_C_. One can readily recognize two demagnetization processes that reveal themselves as the rising components and occur at time scales of subpicoseconds and tens of picoseconds. Therefore, the responses in the general case are described by the expression:


[2] $$\Delta \theta_{\text{K}}\left(\Delta t\right)=\left[A_{\text{r}1}^{\text{K}}\left(1-e^{-\Delta t/\tau_{\text{r}1}^{\text{K}}}\right)+A_{\text{r}2}^{\text{K}}\left(1-e^{-\Delta t/\tau_{\text{r}2}^{\text{K}}}\right)\right]\times e^{-\Delta t/\tau_{\text{d}}^{\text{K}}},$$

where components with amplitudes 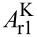 and 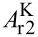 describe the rise (demagnetization), while the factor following the square brackets describes the decay of the signal (magnetization recovery).

**Figure 3:**
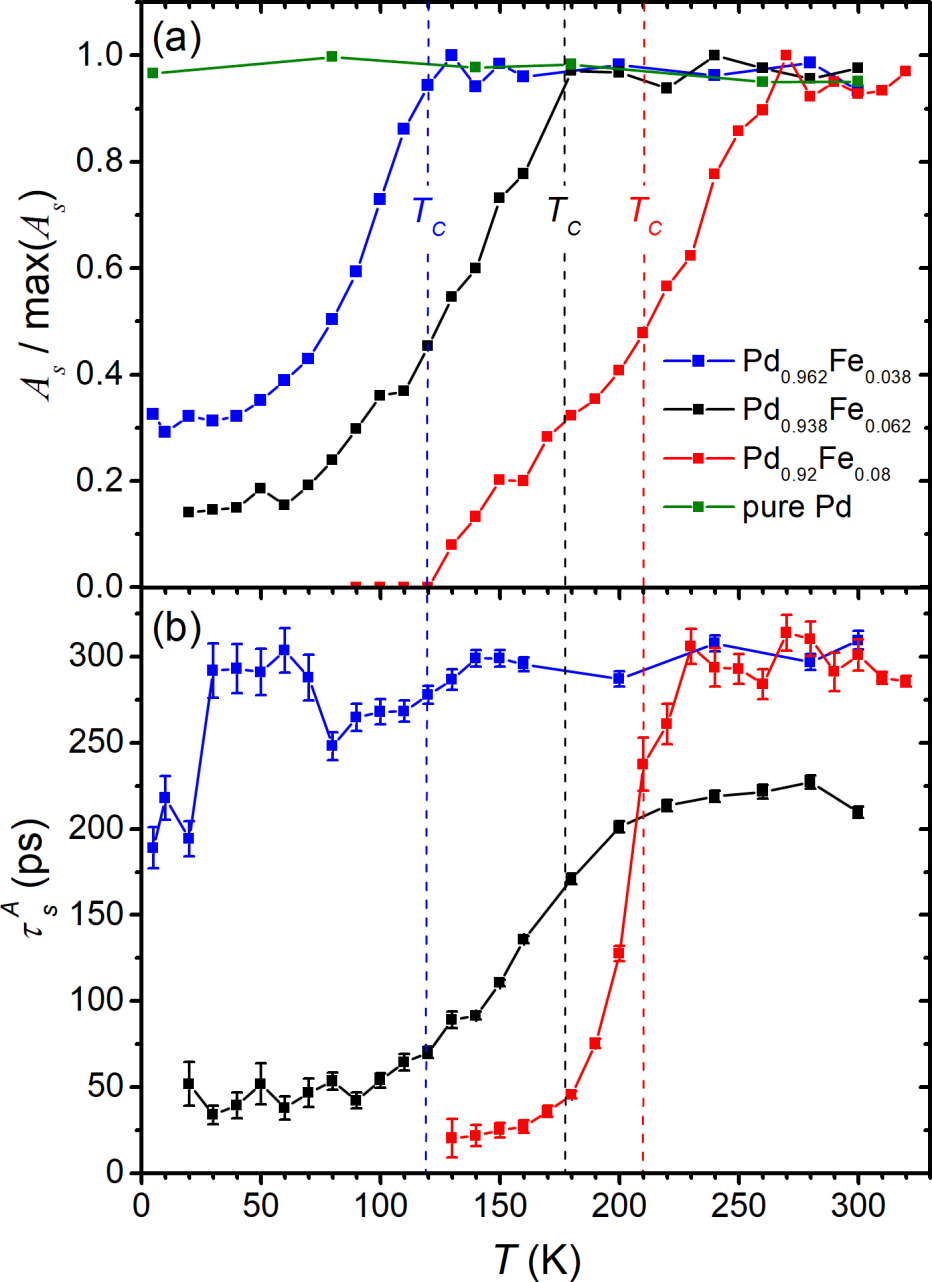
Temperature dependences of the amplitudes (a) and the lifetimes (b) of the slow relaxation components of the reflectivity transients shown in Figure 1. In panel (a) the amplitude *A*_s_ for each sample is normalized to its magnitude at room temperature*.*

**Figure 4:**
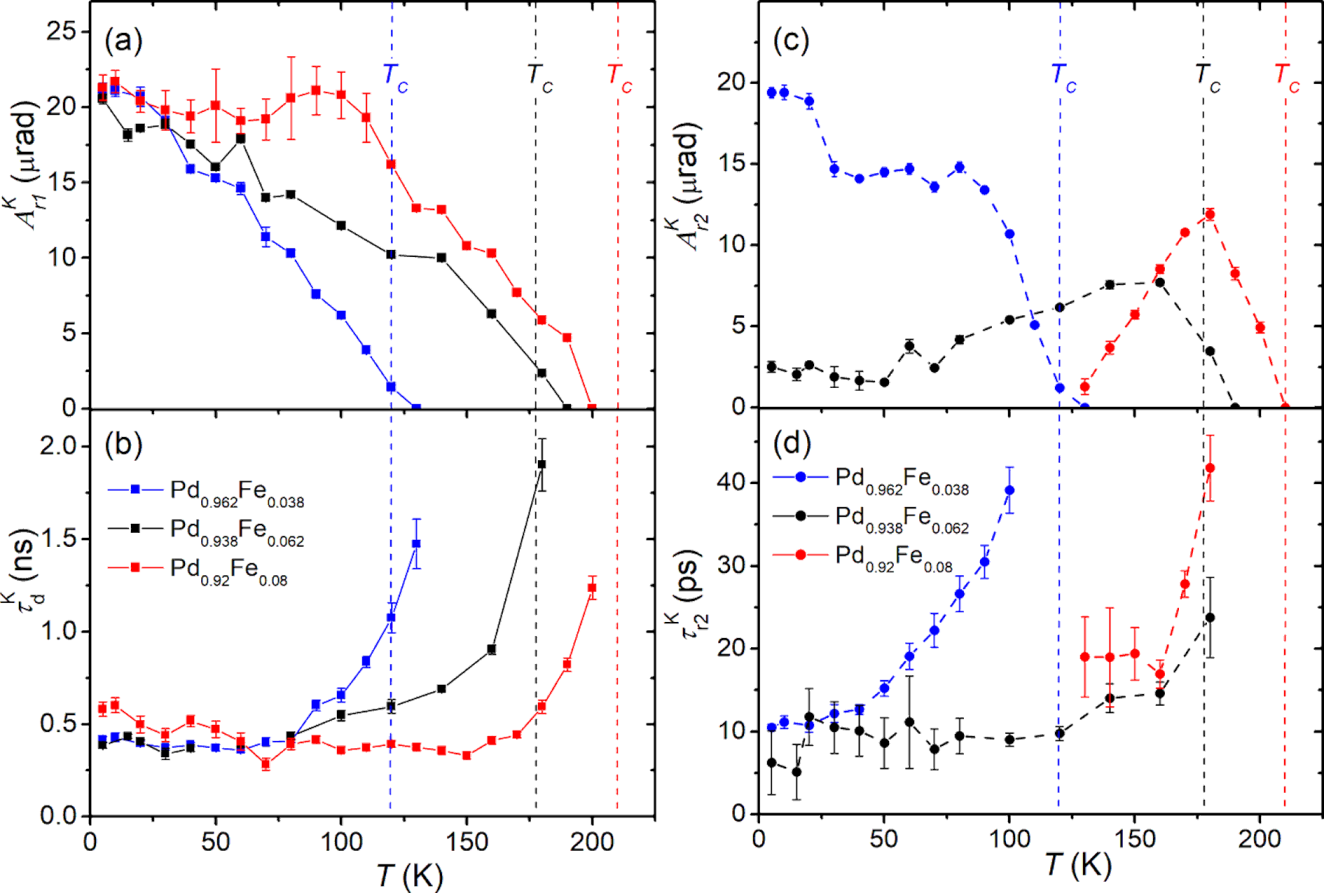
Temperature dependences of the amplitudes of the fast (squares/solid lines) (a) and the slow (circles/dashed lines) (c) photoinduced demagnetization components; characteristic times (b) of the magnetization recovery (squares/solid lines) and (d) of the slow demagnetization (circles/dashed lines) of the Pd_0.962_Fe_0.038_ (blue), Pd_0.938_Fe_0.062_ (black), and Pd_0.92_Fe_0.08_ (red) epitaxial films.

Temperature dependences of the amplitudes and the lifetimes of the selected components, obtained from the fit of the experimental data with Equation 1 and Equation 2, are presented in Figure 3 and Figure 4 for the reflectivity and time-resolved MOKE, respectively. We note here the invariance of the amplitude *A*_s_ (Figure 3a) and relaxation time 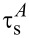 (Figure 3b) at *T* ≥ *T*_C_, and a kink in their dependences at *T* = *T*_C_ for the films with *x* = 0.038 and 0.062. The evolution of this component is not so obvious for the film with *x* = 0.080: The kink in its temperature dependence and the onset of its suppression take place at a temperature slightly above *T*_C_. Below *T*_C_, all three samples reveal a decrease of *A*_s_ and a shortening of 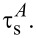 In the samples with 3.8 and 6.2 atom % of iron, the drop of *A*_s_ with the temperature decrease slows down and ceases reaching values of ≈15% and ≈30% of its maximum, respectively, at 5 K.

Other characteristics, that is, the amplitudes *A*_f_ and *B*_s_ and the relaxation times 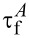 and 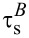, do not reveal any anomalies in their temperature dependences and therefore are not presented. The amplitude of the fast component *B*_f_ for each Pd_1−_*_x_*Fe*_x_* alloy film has a nonzero value practically over the entire temperature range of 5–300 K. It gradually increases with decreasing temperature for samples with 6.2 and 8.0 atom % of iron. For the sample with 3.8 atom % of iron, it has the same behavior down to 150 K, and then decreases to zero at the lowest temperatures. The relaxation time of this component is practically independent of the temperature and is 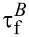 = 0.80 ± 0.10 ps.

Figure 4a shows the temperature dependences of the amplitudes of the fast demagnetization process. It is observed in the entire temperature range below the Curie temperature of the samples. The average rise time of the fast component of demagnetization for all three samples is ≈0.3 ps and depends only slightly on the temperature. The variation with temperature of the amplitude of the slow demagnetization component 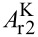 of the Pd_0.962_Fe_0.038_ sample, Figure 4c, is similar in character to that of the fast component. In contrast, in the Pd_0.938_Fe_0.062_ sample, starting from *T*_C_, the amplitude 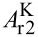 increases with lowering the temperature and reaches a maximum at ≈160 K. On further cooling, the amplitude decreases with a tendency to saturate at a small, but still detectable value at the lowest temperatures. In the Pd_0.92_Fe_0.08_ sample, the slow component is observed only in the range 120 K < *T* < *T*_C_. Here, it also appears at *T*_C_, reaches a maximum at ≈180 K, and drops to zero value at ≈125 K.

Temperature dependences of the characteristic time of the slow demagnetization component are shown in Figure 4d. It has a minimum value for all films at the lowest temperatures of the range of its observation. For the samples with an iron content of 3.8 and 6.2 atom %, the minimum 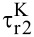 is ≈10 ps, and for a film with 8 atom % of iron, it is ≈20 ps. However, on warming of a sample, the slow demagnetization time increases and becomes several times longer on approaching the Curie temperature.

The magnetization recovery time 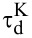 reveals a similar behavior (see Figure 4b) demonstrating a kind of a critical slowing down characteristic for second-order phase transitions. Starting from a value of ≈0.5 ns at the lowest temperatures, 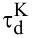 grows rapidly on approaching *T*_C_ of the samples, where it gets two to three times longer.

## Discussion

In this section, we focus our attention at the components of the ultrafast responses of the electronic (reflectivity) and magnetic (Kerr rotation angle) subsystems, which demonstrate a clear correlation with the establishment of the long-range magnetic order in Pd_1–_*_x_*Fe*_x_* films.

**Figure 5:**
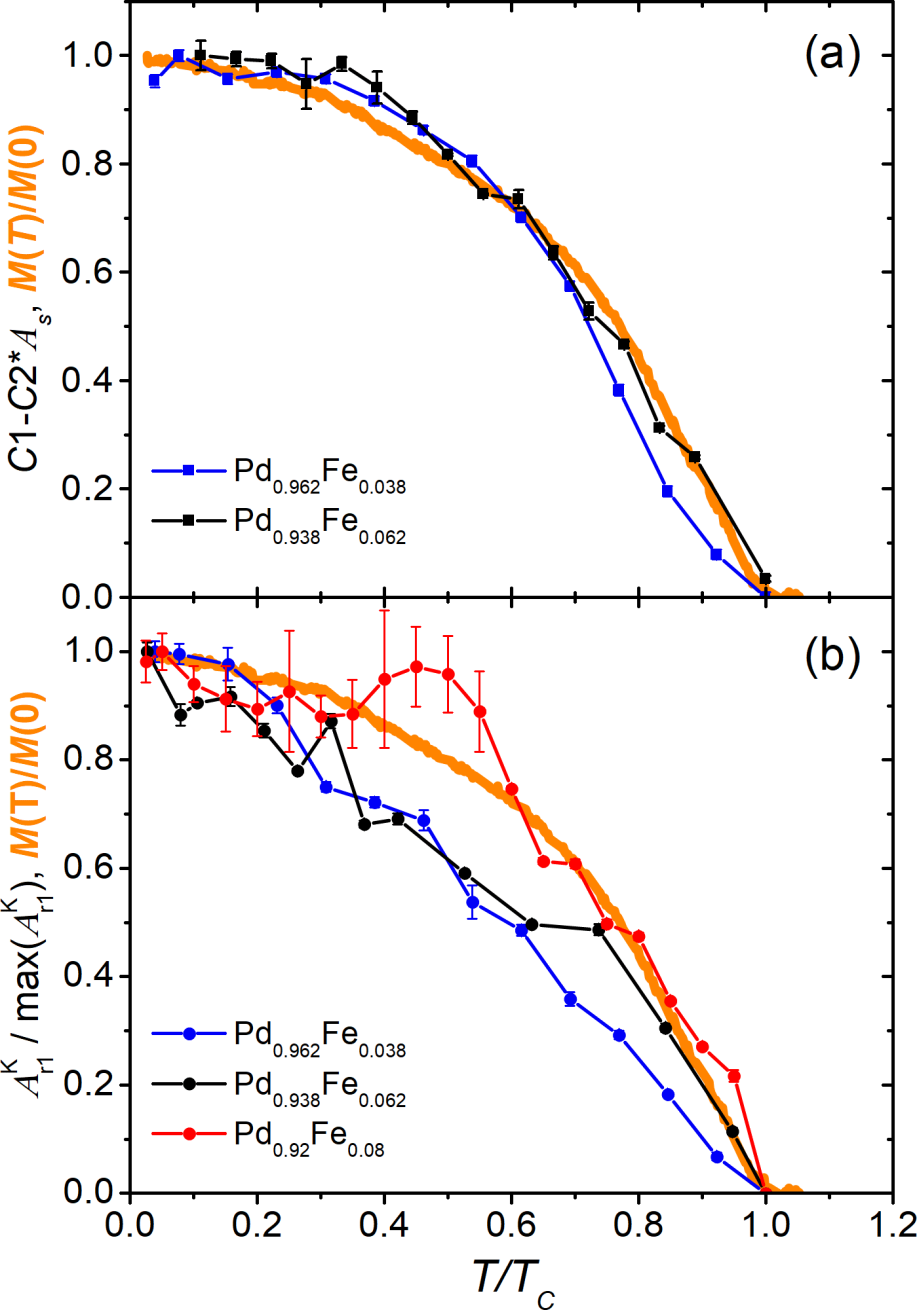
Temperature dependences of the reflectivity slow relaxation amplitudes of the Pd_0.962_Fe_0.038_ (blue) and Pd_0.938_Fe_0.062_ (black) samples, transformed and normalized in magnitude for their comparison with the dependence of the saturation magnetization (thick orange line) (a); the same for the Kerr rotation angle dependences (b).

In the Δ*R*/*R*(Δ*t*) dependences of the alloys, the slow relaxation component with the amplitude *A*_s_ (Figure 3a) follows this trend. While no temperature variation of *A*_s_ is observed for a pure palladium film, a sharp kink close to *T*_C_ towards its decrease appears for the other three samples. Moreover, for the Pd_0.962_Fe_0.038_ film, as well as for the Pd_0.938_Fe_0.062_ film [37], the shape of the properly normalized *A*_s_(*T*) dependence practically reproduces that for the saturation magnetization *M*_s_(*T*)/*M*_s_(0) (Figure 5a). In our opinion, within the framework of the magnetic polaron model [34–35], such a situation can be associated with a decrease in the volume of the paramagnetic phase due to the growth of the fraction of magnetic bubbles.

It is worth noting that the *A*_s_ amplitude for the Pd_0.92_Fe_0.08_ sample vanishes below 120 K. Based on the normalized *A*_s_(*T*) dependences from Figure 3a, one can estimate that in the Pd_0.962_Fe_0.038_ sample, about 30% of the film volume is left in the paramagnetic state at low temperatures; for the Pd_0.938_Fe_0.062_ sample, the volume fraction of the paramagnetic phase is ≈15%. The Pd_0.92_Fe_0.08_ sample is in a homogeneous ferromagnetic state below 120 K. Thus, we associate the slow relaxation of the reflectivity of the Pd_1−_*_x_*Fe*_x_* films with the presence of a residual paramagnetic phase in the sample. We relate its manifestation at temperatures corresponding to the ferromagnetic state of the material to the presence of magnetic inhomogeneities. The latter are most likely formed due to the inhomogeneous distribution of the iron impurities in the palladium host.

As for the Kerr rotation angle dynamics (Figure 2), any detected signals are observed only at temperatures below *T*_C_ for each sample. An interesting feature here is the manifestation of two components in the photoinduced demagnetization, that is, the ultrafast component with a characteristic time of ≈0.3 ps, and a noticeably slower one, occurring on a scale of 10–20 ps. The ultrafast process manifests itself at all temperatures below *T*_C_, and its amplitude grows gradually on cooling. The slower demagnetization component reveals a specific temperature dependence of the amplitude 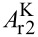, which strongly depends on the composition of the film (see Figure 4a).

Going deeper into the details, the amplitude 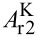 for the sample with *x* = 0.038 increases with decreasing temperature in the entire range of 5 K ≤ *T* < *T*_C_. For samples with *x* = 0.062 and 0.080, this amplitude reaches a maximum rather quickly as the temperature drops below *T*_C_, and then behaves differently for these two samples. In the film with *x* = 0.062, this component gradually decreases to a level of 25–30% of the maximum, without turning to zero down to a temperature of 5 K. In the film with *x* = 0.080, on cooling of the sample below *T*_C_, the amplitude 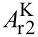 rapidly decreases after reaching the maximum value. It further approaches zero at 120 K and does not recover at lower temperatures. As we can see from Figure 3a and Figure 4a, the observation of the slow component of the demagnetization correlates with the observation of the slow relaxation of the reflectivity (amplitude *A*_s_). In our opinion, this fact makes it possible to relate the slower component of demagnetization with magnetic inhomogeneities in the sample. The amplitude of this component reaches its maximum, apparently, at temperatures corresponding to the maximum degree of magnetic inhomogeneity of the films. The temperature dependence of the subpicosecond demagnetization component clearly correlates with the course of the saturation magnetization of the film (Figure 4a and Figure 5b), and represents, thus, the response of the ferromagnetic component of the films under study.

The origin of the photoinduced demagnetization specific for a magnetically inhomogeneous ferromagnetic/paramagnetic metallic state is important itself. Therefore, it should be discussed explicitly. First, we note that dilute Pd_1−_*_x_*Fe*_x_* alloys are systems in which magnetism is mainly due to the polarization of palladium 4d electrons. The second distinguishing feature of Pd_1−_*_x_*Fe*_x_* alloys is their small spatial scale of magnetic inhomogeneities, which is of the order of 1 nm.

The subpicosecond component of the photoinduced demagnetization is evidently a result of the photoexcitation of the ferromagnetic fraction as it is. It does not demand any process related to the paramagnetic fraction, and therefore we denote it as “on-site demagnetization”. Indeed, such an ultrafast photoinduced demagnetization is a characteristic feature of the 3d metal ferromagnets that has been a matter of intense discussion in past decades [38–43]. An additional demagnetization component with a characteristic time of ≈10 ps requires the presence of a paramagnetic fraction in the material. However, the transfer of the angular momentum between the paramagnetic and ferromagnetic fractions by highly mobile s and p electrons (which occurs due to the s–d interaction [40]) should only increase the rate of photoinduced demagnetization on a subpicosecond scale. This mechanism was justified to explain the ultrafast (subpicosecond) transfer of the angular momentum in F/N heterostructures with large (tens of nanometer) layer thicknesses [40,44–45].

It is our hypothesis that itinerant electron spin diffusion could bring the PM areas into equilibrium with the FM environment and is an origin of the 10 ps transient. Indeed, the diffusion velocity across the length of ≈1 nm on a time scale of 10 ps can be estimated as 10^−9^ m/10^−11^ s = 100 m/s. For the conventional spin diffusion, the spin memory length is 
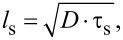
 where 
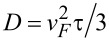
 is the diffusion coefficient, τ_s_ is the Elliott–Yafet spin-relaxation time [46–47], τ is the charge transport relaxation time, and *v*_F_ is the Fermi velocity. For the purpose of order-of-magnitude estimation we define the spin-diffusion velocity *v*_s_ as 
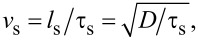
 from which 
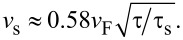
 Modern band-structure calculations [48–49] show that more than 95% of the electron density of states at the Fermi energy comes from the itinerant 4d electrons. The Fermi velocity of 3d electrons in iron-group ferromagnetic metals was a subject of interest in magnetic nanostructures [50–52] and had a value of about 3 × 10^5^ m/s. Being stronger localized in the narrower 4d bands [44], the itinerant 4d electrons must have a lower velocity, say 10^5^ m/s. Then, with the transport time τ ≈ 10^−14^ s and the electron-spin relaxation time τ_s_ ≈ 10^−9^ s [53] we get *v*_s_ ≈ 1.8 × 10^2^ m/s as an upper bound. An order-of-magnitude matching of the obtained *v*_s_ value with the initial guess makes the 4d electron spin diffusion a plausible mechanism of the observed 10–25 ps demagnetization component in a mixed PM/FM state in the palladium-rich PdFe alloys.

## Conclusion

Based on the analysis of the experimental data on ultrafast optical and magneto-optical spectroscopy in comparison with the magnetometry data, responses have been identified inherent to the magnetically inhomogeneous state of the epitaxial Pd_1−_*_x_*Fe*_x_* alloy films. The vanishing of these components with decreasing temperature makes it possible to establish a lower limit for the concentration of iron in palladium and the operation temperature that ensures the magnetically homogeneous ferromagnetic state of the films. This is one of the key conditions for their use as weak links in magnetic Josephson junctions and superconducting memory elements based on spin valves.
